# Hydration Responses to Pre-Exercise Sodium Hyperhydration at Rest and During Cycling in the Heat and Across Menstrual Cycle Phases

**DOI:** 10.3390/nu17233672

**Published:** 2025-11-24

**Authors:** Lilia Convit, Liliana Orellana, Julien D. Périard, Amelia J. Carr, Stuart Warmington, Ashwin K. V. Mruthunjaya, Angel A. J. Torriero, Rhiannon M. J. Snipe

**Affiliations:** 1 Centre for Sport Research, School of Exercise and Nutrition Sciences, Deakin University, Burwood, VIC 3125, Australia; lilia.convitcordova@deakin.edu.au (L.C.); amelia.carr@deakin.edu.au (A.J.C.); r.snipe@deakin.edu.au (R.M.J.S.); 2 Biostatistics Unit, Deakin University, Geelong, VIC 3220, Australia; l.orellana@deakin.edu.au (L.O.); 3 Research Institute for Sport and Exercise, University of Canberra, Bruce, ACT 2617, Australia; julien.periard@canberra.edu.au (J.D.P.); 4 Institute for Physical Activity and Nutrition, Deakin University, Burwood, VIC 3125, Australia; stuart.warmington@deakin.edu.au (S.W.); 5 School of Life and Environmental Sciences, Deakin University, Burwood, VIC 3125, Australia; a.varamballimruthunjaya@deakin.edu.au (A.K.V.M.); angel.torriero@deakin.edu.au (A.A.J.T.)

**Keywords:** dehydration, fluid balance, thermoregulation, hypervolemia, arginine vasopressin, progesterone, menstrual cycle phase, female athletes

## Abstract

**Purpose**: This study examined hydration responses to sodium hyperhydration in female athletes at rest and during cycling across the early follicular and mid-luteal phases of the menstrual cycle. **Methods**: Twelve cyclists/triathletes consumed 30 mL·kg^−1^ fat-free mass fluid with either sodium chloride (7.5 g·L^−1^) or placebo (sucrose) 2 h before 75 min of steady-state cycling (60% VO_2peak_) and a 200 kJ time trial (TT) in a hot environment (34 °C, 60% RH). Body mass was measured, and urine was collected every 30 min, whilst blood samples were taken hourly pre-exercise, post-steady-state, and post-TT. **Results**: During pre-exercise, sodium hyperhydration increased fluid retention (509.0 mL, 95% CI: [349.0, 669.0]; *p* < 0.001), while reducing urine volume (−107.4 [−146.7, −68.1] mL; *p* < 0.001). During exercise, body mass loss was lower with sodium during steady-state (−0.20%; *p* = 0.001) and TT (−0.15%; *p* = 0.037), but sweat rates were reduced with sodium only during steady-state (−0.08 L·h^−1^; *p* = 0.001). Exploratory analyses showed greater effects in the early-follicular phase, with reductions in body mass loss (−0.26%; *p* = 0.004), sweat rate (−0.1 L·h^−1^; *p* = 0.003), and post-TT arginine vasopressin (−10.8 [−19.2, −2.3] pg·mL^−1^; *p* = 0.013). **Conclusions**: Sodium hyperhydration effectively enhanced fluid retention and reduced body mass loss during exercise in the heat. While no consistent main effects of menstrual cycle phase were observed, some phase-specific differences emerged, particularly in the early-follicular phase. These findings highlight the importance of monitoring hydration responses across the menstrual cycle and tailoring strategies to individual needs, rather than applying universal phase-specific recommendations.

## 1. Introduction

Achieving euhydration and maintaining appropriate hydration status (e.g., avoiding hypohydration and hyponatraemia) is critical for health and athletic performance, particularly during prolonged exercise in hot and/or humid conditions [1]. During exercise under heat stress, euhydration mitigates thermal strain (i.e., rise in core and skin temperature [2]) and cardiovascular strain (e.g., elevated heart rate for a given workload [3]) compared with hypohydration. It also supports sweat production and evaporative cooling during exercise, attenuating the increase in heat storage and fatigue exacerbation [3]. However, maintaining adequate hydration presents considerable challenges for endurance athletes, particularly during exercise in the heat [4,5,6]. For example, body mass losses ≥ 2% are commonly observed even with voluntary fluid intake, which can lead to electrolyte disturbances (e.g., hypo- and hypernatremia) [3].

A useful strategy to mitigate dehydration during exercise in the heat is pre-exercise hyperhydration using sodium, an osmotically active ingredient [7,8], known to enhance fluid retention and, therefore, temporarily increase total body water above euhydration [9]. Sodium hyperhydration elevates plasma osmolality and volume [6,10], promoting extracellular fluid retention [10,11,12] by enhancing sodium reabsorption in the kidney while reducing urine output [11]. This physiological process is regulated, in part, by the arginine vasopressin (AVP) hormone, which acts on osmoreceptors to stimulate water reabsorption in the kidney tubules, thereby conserving total body water [13]. Previous research confirmed that sodium hyperhydration delays the onset of dehydration and decreases cardiovascular and thermal strain [14] during exercise in temperate and hot conditions, subsequently improving endurance work capacity and performance [15,16]. However, responses to sodium hyperhydration may depend on individual factors, such as training status, hydration habits, and sex hormones, particularly in female athletes [17,18].

Despite the growing interest in hydration strategies, research on hydration outcomes remains heavily skewed toward male participants, with females representing ~30% of participants in recent hydration studies [18]. Variations in female ovarian hormones (e.g., oestrogen and progesterone) across the menstrual cycle can influence renal function, fluid and electrolyte balance, and hydration status, potentially affecting fluid retention in response to sodium hyperhydration and exercise performance in the heat [19,20]. The higher oestrogen concentration of the late follicular phase of the menstrual cycle leads to increased plasma volume and AVP secretion [21]. In contrast, a combination of moderate oestrogen and high progesterone concentrations in the mid-luteal phase activates the renin–angiotensin–aldosterone system to enhance sodium and water reabsorption [21,22]. These menstrual cycle phase-specific hormonal influences on body water may affect hydration responses at rest following sodium hyperhydration. Additionally, increased fluid retention in the mid-luteal phase, when combined with excessive fluid intake, may pose a greater risk for exercise-associated hyponatraemia [23].

Previous research has demonstrated that sodium hyperhydration improves time to exhaustion (TTE) [16] and time trial (TT) performance [24] in female athletes, yet hydration responses at rest and during exercise across the menstrual cycle remain largely unexplored. Most existing studies have either examined hydration under resting conditions only [9,11,25,26], investigated exercise capacity (TTE) [16,27] or focused on a single menstrual cycle phase, or included mixed cohorts with oral contraceptive users, which do not reflect natural hormonal variations [16,18].

To date, no study has comprehensively assessed hydration outcomes, such as fluid retention, plasma volume, and hormonal responses, both at rest and during exercise (steady-state cycling and TT) across menstrual cycle phases in naturally menstruating females. A clearer understanding of these potential phase-specific hydration responses to sodium hyperhydration at rest and in response to exercise is required for developing precise, individualised fluid intake guidelines to help refine hydration responses and improve performance in female athletes. This has both academic and practical significance. Academically, the study helps close a long-standing evidence gap in female-specific hydration physiology, advancing understanding of how endogenous hormones modulate fluid regulation and thermoregulatory responses. It also enhances the ecological validity of female-focused research and provides a foundation for future mechanistic studies beyond generalised male-derived assumptions. Practically, identifying whether sodium hyperhydration responses differ across menstrual cycle phases will inform applied hydration planning and improve precision in pre-event preparation, supporting both performance and heat safety for female athletes competing in warm environments.

Therefore, this study aimed to investigate the effect of pre-exercise sodium hyperhydration on hydration measures and markers in female athletes at rest and in response to cycling in the heat across the early-follicular and the mid-luteal phases of the menstrual cycle. It was hypothesised that sodium hyperhydration would enhance fluid retention and plasma volume at rest, and attenuate thermal and cardiovascular strain during exercise in the heat, with more pronounced effects observed in the mid-luteal phase due to progesterone- and oestrogen-mediated activation of fluid-regulatory mechanisms.

## 2. Materials and Methods

### 2.1. Participants and Recruitment

Twelve endurance-trained female cyclists/triathletes (age 33 ± 5 years, height 165 ± 7 cm, body mass 62.3 ± 6.3 kg, fat-free mass 46.3 ± 4.4 kg, VO_2peak_ 53.5 ± 7.8 mL·kg^−1^·min^−1^), classified based on athletic calibre [28] as Tier 2 (*n* = 9) and Tier 3 (*n* = 3), completed the study. Eligibility criteria included a minimum of two years of cycling experience, regular training of more than three sessions (>5 h per week [29]), good general health (e.g., free of chronic conditions), and having a natural menstrual cycle (21–35 days [17]). Exclusion criteria included any regular exposure to high temperatures (>25 °C) within the previous two months, pregnancy, breastfeeding, or discontinuation of hormonal contraceptive use in the previous six months. Screening and informed consent were conducted using REDCap (version 14.0.29, Vanderbilt University).

### 2.2. Experimental Design and Procedures

#### 2.2.1. Design

This study employed a randomised, double-blinded (for the supplement but not the menstrual cycle phase), cross-over design with four sessions, defined by the combination of treatment (sodium chloride or placebo) and menstrual cycle phase (early follicular or mid-luteal). A quasi-balanced randomisation was used to distribute starting treatment and phase as evenly as possible while accommodating menstrual cycle phase scheduling. Across the first sessions, seven participants began with NaCl and six with a placebo. Seven began in the early follicular phase and six in the mid-luteal phase. The subsequent session order followed the randomisation list, subject to each participant’s phase timing and availability, so sequences were not uniform across all participants.

#### 2.2.2. Participants

Participants completed one familiarisation session followed by four experimental sessions. Experimental sessions occurred during the cooler months in Melbourne, Victoria (April–October ~16.2 ± 1.6 °C [30]) to avoid natural heat acclimatisation. Only one familiarisation session was conducted to reduce participant burden. The early-follicular (3–6 days after the onset of menstruation) and the mid-luteal (7–9 days after positive urinary luteinising hormone test [31,32,33]) phases of the menstrual cycle were confirmed using the three-step method (i.e., calendar-based counting, urinary luteinising hormone testing, and retrospective serum [17-β-oestradiol] and [progesterone]). Additional details on participant recruitment, menstrual cycle tracking and previously reported data on performance outcomes, thermal responses (rectal and skin temperature), heart rate, and perceptual measures (gastrointestinal symptoms, thirst, rating of perceived exertion, and thermal sensation) are available in a prior publication [24].

#### 2.2.3. Familiarisation Session

Participants’ height (m; Holtain Limited, Crymych, UK), body mass (kg; TANITA Ingleburn, Australia), body fat (%), and fat-free mass (kg; Lunar iDXA; GE Healthcare, Waukesha, WI, USA) were measured to determine appropriate fluid and sodium dosage. Participants then performed a progressive incremental cycling test to exhaustion in temperate laboratory conditions (~21 °C; ~40% relative humidity (RH)) to determine VO_2peak_ [34] and the exercise intensity for steady-state cycling during the experimental sessions. The test was completed on a LODE bike (Excalibur Sport, Groningen, The Netherlands) using a calibrated Moxus Modular Metabolic cart (AEI Technologies, Inc., Bastrop, TX, USA). After a 30 min rest, participants completed one familiarisation session of a simulated 200 kJ cycling TT in temperate conditions.

#### 2.2.4. Experimental Sessions

Participants were provided with a frozen dinner (2236 kJ, 13 g protein, 15 g total fats, 60 g carbohydrates, 883 mg sodium) for consumption the night before testing and instructions on how to complete the food and exercise diary using a smartphone app (Easy Diet Diary, Xyris Pty Ltd., Brisbane, Australia). They were advised to continue their typical diet throughout the study and to record all food, fluid, and caffeine intake, as well as exercise mode, duration, and intensity, for the 24 h preceding each experimental session. Dietary and fluid intake were extracted from the diaries and analysed using a dietary analysis software (FoodWorks Professional, v10.0, Xyris Pty Ltd., Brisbane, Australia) to verify consistency in pre-trial hydration and dietary intake across sessions. Participants were required to abstain from alcohol, high-sodium food, and fluids (to avoid confounding factors for sweat [Na^+^] and plasma volume [35]), and vigorous exercise before each experimental session. Participants attended the laboratory at the same time of day, rested and overnight fasted (~10 h), and consumed a standardised breakfast (~1857 kJ, 11 g protein, 13 g total fats, 66 g carbohydrates, 306 mg sodium) with 250 mL of water, approximately 30 min before commencing the hyperhydration protocol. Habitual caffeine drinkers consumed a standardised coffee sachet (~50 mg caffeine) with water to avoid withdrawal symptoms [36].

##### Hyperhydration Protocol

The hyperhydration protocol was completed at rest two hours before the commencement of exercise and consisted of hyperhydration with sodium (7.5 g·L^−1^ of sodium chloride in gelatine pills with 30 mL·kg^−1^ fat-free mass of water with regular sucrose-containing cordial (~17% sucrose; ~10.2 g; Cottee’s Cordials, Cadbury Schweppes, Melbourne, Australia)) or sodium-free placebo (matched number of pills filled with table sugar and matched sucrose-free cordial (same flavour)) to match carbohydrate intake between intervention and placebo. All drinks and pills were prepared, coded, and randomised by an independent researcher not involved in data collection or analysis, to ensure double blinding of participants and investigators. Fluid volume and pills were divided equally (using opaque paper bags) into four 20 min time points (T-120, T-100, T-80, and T-60 before exercise). At each time point, participants consumed one-quarter of the assigned fluid together with one-quarter of the capsules within five minutes.

##### Exercise Protocol

After the hyperhydration protocol, participants walked for ~2 min to an environmental chamber set to 34 °C and 60% RH, with 4.5 m·s^−1^ airflow (Kestrel Instruments, Boothwyn, PA, USA) and completed 75 min of steady-state cycling at 60% of their VO_2peak_ power output (no fluid ingested). Workloads were standardised within participants across all four trials (mean absolute power: 111 W, range 66–166 W). This was followed by a 10 min break in the environmental chamber to obtain blood samples and nude body mass measurement, and for participants to consume ~7 mL·kg^−1^ fat-free mass of temperate water. Participants then completed a 200 kJ cycling TT (mean duration: 23.1 min; range: 14.3–36.0 min) [37,38]. No verbal encouragement was provided except for a time countdown during the first 75 min of steady-state cycling and a countdown of work performed every 50 kJ during the TT.

#### 2.2.5. Blood, Urine and Sweat Analysis

**Figure 1:**
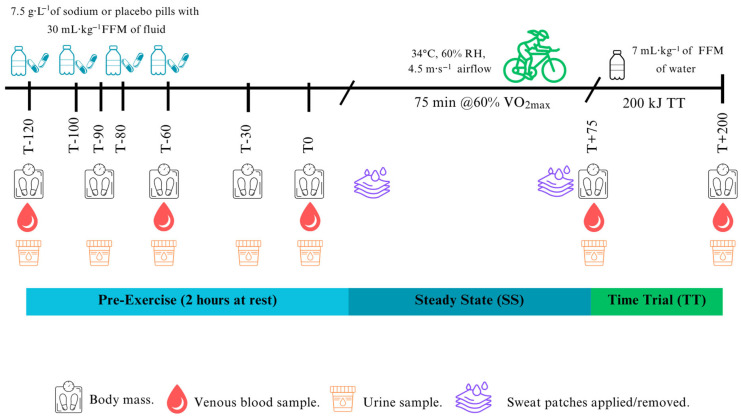
Schematic of the experimental sessions.

Blood samples were collected in a semi-recumbent position via aseptic cannulation (McFarlane Medical, Victoria, Australia) in the antecubital vein pre-exercise (T-120, T-60, T0), during exercise (T+75) and post-exercise (T+200 kJ) (~16 mL at baseline and ~12 mL at other timepoints on each visit). At all sampling timepoints, participants were seated with their arms supported on a phlebotomy chair at a consistent height. For the during- and post-exercise samples, participants dismounted the ergometer and were seated in a chair positioned next to it to maintain the same posture during both collections. Posture, arm height, and timing were matched within participants across all visits to control for posture-related plasma volume shifts. One millilitre of blood was drawn into a safePICO syringe (Radiometer, Copenhagen, Denmark) and analysed in duplicate for blood [Na^+^], haematocrit [Hct], and haemoglobin [Hb] using an ABL800Flex Blood-Gas Analyser (Radiometer, Copenhagen, Denmark; inter-analytical coefficient of variation (CV) 0.8%). The residual blood was centrifuged (3600 rpm, 4 °C, 10 min, and serum (6 mL) left to clot for 30 min). Fifty microlitres of plasma were analysed in duplicate via freezing point depression (Osmometer K-7400S, Knauer, Germany; CV 2.4%) for plasma osmolality. Residual plasma and serum were stored at −80 °C until ELISA kit analysis. Plasma AVP was measured using Abcam’s extraction protocol, with an additional freeze-drying step to concentrate the samples and improve detectability. Plasma samples were snap-frozen in liquid nitrogen and freeze-dried for ~24 h using an SP Scientific BenchTop Pro freeze dryer (SP Industries, Warminster, PA, USA). Lyophilisation was conducted under vacuum conditions (−50 °C and ≤0.1 mbar) until complete dehydration was achieved. The dried samples were then reconstituted in ELISA assay buffer according to the manufacturer’s instructions before analysis (Abcam, UK; CV 8.4%). This additional freeze-drying step was necessary, as the kit’s lower detection limit was insufficient to quantify AVP in the original samples. Serum [17-β-oestradiol] (CV 2.2%) and [progesterone] (CV 4.1%) concentrations were analysed as per the manufacturer’s instructions (Abcam, UK). Nude body mass (kg) was measured every 30 min pre-exercise, post-steady-state, and post-TT (kg; TANITA Wedderburn Australia). During pre-exercise (T-120, T-90, T-60, T-30, T0), exercise (T+75), and post-exercise (T+200 kJ), participants provided a urine sample using a 24 h container (Greiner Vacuette, Interpath, Australia) to measure urine volume (mL), urine specific gravity (USG; PAL-10S, Atago U.S.A., Inc., Tokyo, Japan), urine osmolality (mOsm·kgH_2_O^−1^; CV 1.8%), and urine sodium [Na^+^] (ppm, LAQUAtwin Na^+^ pocket meter, HORIBA Scientific, Irvine, CA, USA; CV 5.6%) (Figure 1). After the visible onset of sweating (approximately 5 min into exercise, as determined by visual inspection to ensure active perspiration before patch placement), the skin at each anatomical site was cleaned with an alcohol wipe, followed by a distilled water wipe and then tap-dried before patch application. Sterile absorbent pads (3M TM, St. Paul, MI, USA) were applied at four sites: chest (superior to the nipple and 5 cm lateral from the sternum), scapula (7 cm lateral from the vertebral column), forearm (mid-dorsal), and thigh (mid-ventral). Pads were monitored continuously during exercise and left in place for a maximum of 60 min, but they were removed earlier if there was a risk of saturation. Upon removal with sterile tweezers, pads were placed into sterile syringes for immediate analysis of sweat sodium concentration [Na^+^] (HORIBA Scientific, Irvine, CA, USA; CV 3.9%) [39].

#### 2.2.6. Derived Variables

Fluid retention (mL) was calculated as the difference between fluid ingested and urine volume excreted, and the percentage of ingested fluid retained was derived accordingly [40]. Change in plasma volume was calculated based on haemoglobin and haematocrit values using the method of Dill and Costill [41], without applying a correction factor. Sweat loss and hourly sweat rate (L·h^−1^) were determined from changes in body mass, accounting for fluid intake and urine output [42]. Mean whole-body sweat sodium concentration was estimated using regional sweat [Na^+^] data as previously described [43].

### 2.3. Sample Size Estimation and Justification

This is an analysis of secondary outcomes from a previously published paper [24]. The original sample size calculations were based on the trial’s primary outcome. So, results should be considered exploratory in nature.

### 2.4. Statistical Analysis

Outcomes were analysed using linear mixed models (LMM) with participants as a random effect to account for repeated measures [44]. Three time periods were considered: pre-exercise hyperhydration (T-120 to T0 min), steady-state (T+75), and TT (T+200 kJ). For outcomes with one observation per session, the LMM included ‘intervention’ and ‘phase’ and their interaction as fixed effects. From these models, we reported the marginal means for intervention and placebo, (a) overall (across both menstrual cycle phases) and (b) within menstrual cycle phases. For longitudinal outcomes with more than one observation per session, the LMM included ‘intervention’, ‘phase’, and ‘time’ (categorical), and all interactions as fixed effects. From these models, we reported the marginal means for intervention and placebo averaged across the period (a) overall (both menstrual cycle phases), (b) within the menstrual cycle phases, and (c) plots of the estimated means across time for each combination of ‘intervention’ and ‘phase’ (Stata/SE version18, StataCorp, College Station, TX, USA). Due to only four out of 12 participants providing urine samples during exercise (T+75) and post-exercise (T+200 kJ), these time points were excluded from statistical analysis. AVP concentrations were calculated using the software package Origin 8.0 (OriginLab, Northampton, MA, USA) by fitting a four-parameter logistic (4PL) curve to the optical density values of known standards. All results are reported as estimates and 95% confidence interval (CI), except for participants’ characteristics, which are reported as mean ± standard deviation (SD). Statistical significance was defined as *p* < 0.05.

All participants (*n* = 12) were included in the primary analysis. A sensitivity analysis was performed on the No-LPD subgroup (*n* = 8) to assess the impact of excluding those with potential luteal phase deficiency (LPD) (*n* = 4), defined by progesterone levels < 16 nmol·L^−1^ [17], while data from all participants in the early-follicular phase was retained.

## 3. Results

### 3.1. Participants

All experimental sessions were completed during the early-follicular phase (day 5 ± 1) and the mid-luteal phase (day 22 ± 3) of the menstrual cycle. Eight out of twelve participants had progesterone concentrations > 16 nmol·L^−1^ and were classified as the No-LPD group, while four participants were identified as having potential luteal phase deficiency (LPD group) and were excluded from the sensitivity analysis. No significant differences were observed in participant characteristics (age, height, body mass, fat-free mass, VO_2peak_) between the No-LPD and potential LPD participants (Table 1) except for progesterone concentrations (early-follicular phase: 5.3 [4.2, 6.4] vs. 2.7 [1.1, 4.3] nmol·L^−1^, *p* = 0.009; mid-luteal phase: 73.6 [63.4, 83.9] vs. 17.5 [3.5, 31.5] nmol·L^−1^, *p* < 0.001).

The interactions intervention × menstrual cycle phase × time and intervention × menstrual cycle phase were not significant for all outcomes during pre-exercise hyperhydration, steady-state, or TT. Therefore, only the intervention effects for all participants (overall) and within each menstrual cycle phase are reported (Table 2).

### 3.2. Baseline

There were no significant differences between intervention arms or within menstrual cycle phases for any outcome at baseline (Table 2). All participants arrived well-hydrated, with a USG ≤ 1.016 au.

### 3.3. Pre-Exercise Hyperhydration (At Rest)

**Figure 2:**
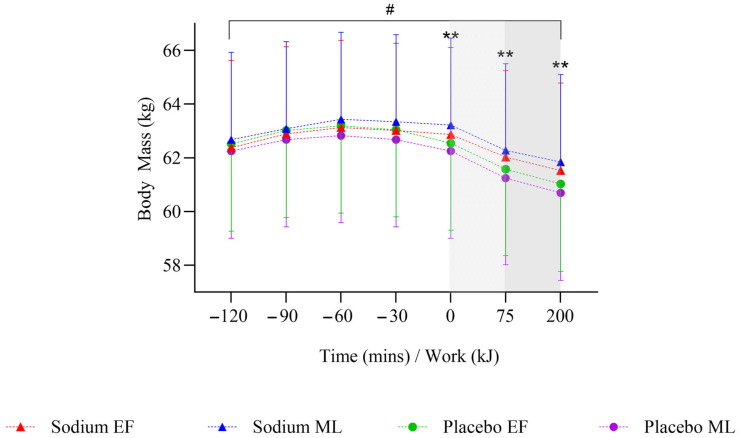
Mean change in body mass after two hours of hyperhydration at rest, during the steady state cycling (75 min), and the 200 kJ cycling time trial in the heat, with sodium and placebo hyperhydration across the early-follicular and mid-luteal phases of the menstrual cycle (*n* = 12). Linear mixed model included ‘intervention’, ‘phase’, and ‘time’ (categorical) and all possible interactions as fixed effects. ** *p* < 0.001 between sodium and placebo regardless of menstrual cycle phase. # *p* < 0.05 between sodium and placebo in the mid-luteal phase of the menstrual cycle. Shaded regions denote steady-state cycling (light grey) and the 200 kJ time-trial (dark grey).

**Figure 3:**
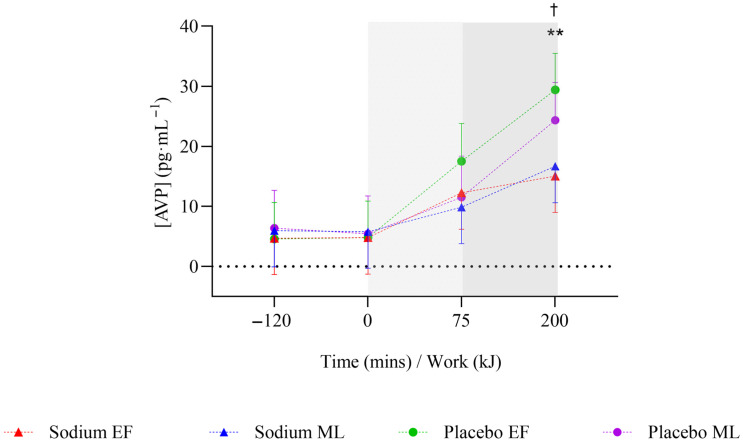
Mean change in arginine vasopressin hormone concentration after two hours of hyperhydration at rest, during the steady state cycling (75 min), and the 200 kJ cycling time trial in the heat, with sodium and placebo hyperhydration across the early-follicular and mid-luteal phases of the menstrual cycle (*n* = 12). EF: early follicular phase; ML: mid-luteal phase. Linear mixed model included ‘intervention’, ‘phase’, and ‘time’ (categorical) and all possible interactions as fixed effects. ** *p* < 0.001 between sodium and placebo, regardless of menstrual cycle phase. † *p* < 0.05 between sodium and placebo in the early-follicular phase of the menstrual cycle. Shaded regions denote steady-state cycling (light grey) and the 200 kJ time-trial (dark grey).

During the pre-exercise hyperhydration period (T-120 to T0), there was no significant difference in body mass between sodium and placebo (*p* = 0.096). However, it was higher with sodium compared to placebo in the mid-luteal phase (early-follicular phase: −0.01 [−0.51, 0.49] kg; mid-luteal phase: 0.62 [0.1, 1.13] kg; Figure 2). Compared to placebo, sodium hyperhydration increased fluid retention (overall: 509 [348.7, 669.3] mL; early-follicular phase: 415.3 [191.6, 638.9] mL; mid-luteal phase: 602.7 [372.9, 832.4] mL) and percentage of ingested fluid retained (overall: 36.9 [25.1, 48.7]%; early-follicular phase: 29.7 [13.3, 46.2]%; mid-luteal phase: 44.1 [27.2, 61]%), with higher values observed in the mid-luteal phase (all *p* < 0.001; Table 2). USG (0.002 [0.001, 0.003] au; *p* < 0.001), urine osmolality (91.9 [50.6, 133.3] mOsm·kgH_2_O^−1^), and urine sodium (451.5 [269.9, 633.2] ppm) were significantly higher, and urine volume significantly lower (−107.4 [−146.7, −68.1] mL) with sodium compared to placebo, and this was observed in both menstrual cycle phases (all *p* < 0.05; Table 2). There were no significant differences overall or within menstrual cycle phases in AVP concentration during pre-exercise hyperhydration between sodium and placebo (Figure 3).

**Figure 4:**
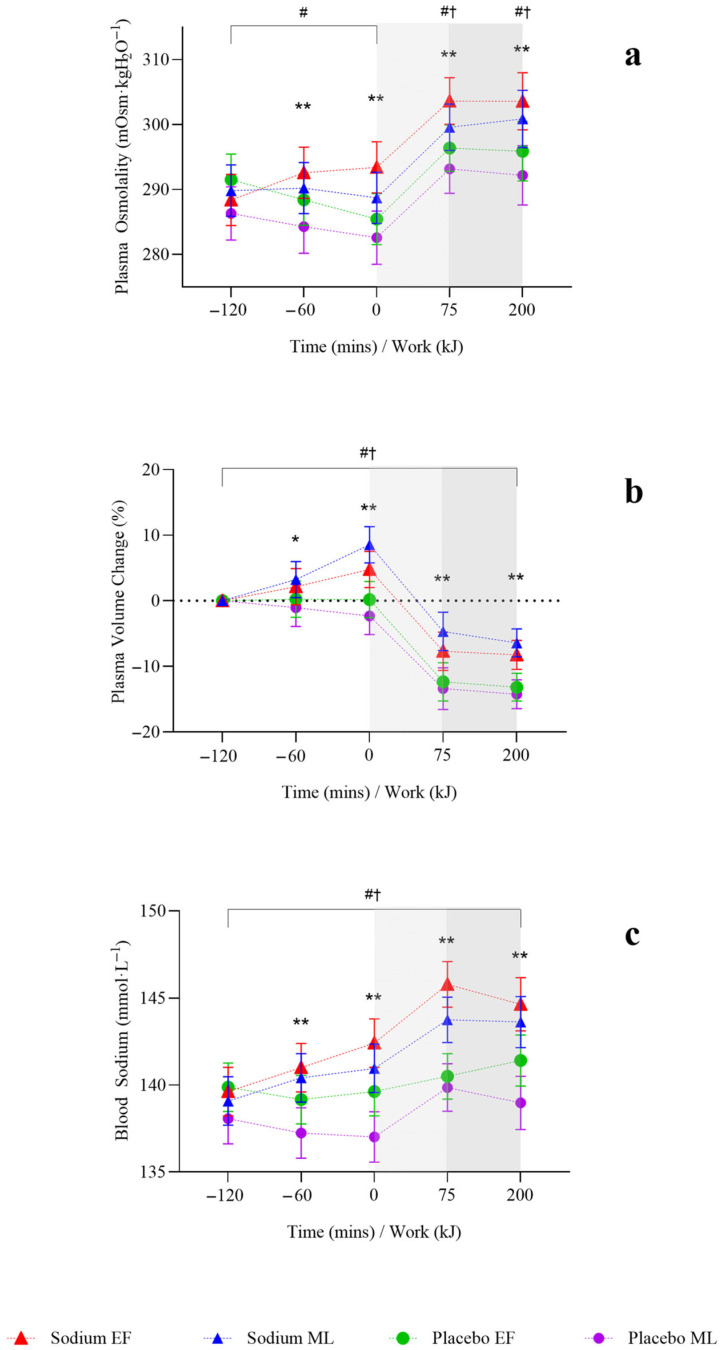
Hydration responses after two hours of hyperhydration at rest, during the steady state cycling (75 min), and the 200 kJ cycling time trial in the heat, with sodium and placebo hyperhydration across the early-follicular and mid-luteal phases of the menstrual cycle (*n* = 12). (**a**), Plasma osmolality; (**b**), plasma volume change; (**c**), blood sodium. EF: early follicular phase; ML: mid-luteal phase. Linear mixed model included ‘intervention’, ‘phase’, and ‘time’ (categorical) and all possible interactions as fixed effects. * *p* < 0.05 and ** *p* < 0.001 between sodium and placebo, regardless of menstrual cycle phase. † *p* < 0.05 between sodium and placebo in the early-follicular phase of the menstrual cycle. # *p* < 0.05 between sodium and placebo in the mid-luteal phase of the menstrual cycle. Shaded regions denote steady-state cycling (light grey) and the 200 kJ time-trial (dark grey).

Plasma osmolality increased overall with sodium compared to placebo (4 [2, 7] mOsm·kgH_2_O^−1^; *p* = 0.002) but was observed only in the mid-luteal phase (Figure 4a). Plasma volume change (3.62 (2.13, 5.11]%; *p* < 0.001; Figure 4b) and blood sodium (2 [1, 3)] mmol·L^−1^; *p* < 0.001; Figure 4c) were significantly higher with sodium compared to placebo, and this was observed in both menstrual cycle phases.

### 3.4. Steady State

On completion of steady-state (T+75), body mass was significantly higher with sodium compared to placebo (0.74 [0.36, 1.13] kg; *p* < 0.001), but this was observed only in the mid-luteal phase (Figure 2). Body mass loss (−0.2 [−0.33, −0.08]%) and sweat rate (−0.08 [−0.13, −0.03] L·h^−1^) were significantly lower with sodium compared to placebo (both *p* = 0.001), but this was observed only in the early-follicular phase. There were no significant differences in sweat sodium concentration or AVP concentration between sodium and placebo or within menstrual cycle phases (Table 3, Figure 3).

Plasma osmolality (7 [3.0, 10.0] mOsm·kgH_2_O^−1^; *p* < 0.001) and blood sodium (5 [3.0, 6.0] mmol·L^−1^; *p* < 0.001; Figure 4a,c) were significantly higher with sodium compared to placebo, and this was observed in both menstrual cycle phases. Plasma volume decreased less during the steady-state (T+75) with sodium compared to placebo overall (6.69 [3.82, 9.56]%; *p* < 0.001; Figure 4b) and in both menstrual cycle phases.

### 3.5. Time Trial

On completion of TT (T+200 kJ), body mass was significantly higher with sodium compared to placebo (0.82 [0.42, 1.22] kg; *p* < 0.001), and this was observed only in the mid-luteal phase (Figure 2). Body mass loss (−0.15 [−0.28, −0.01]%) was significantly lower with sodium compared to placebo, with no differences observed within menstrual cycle phases (Table 3). There were no significant differences in sweat rate between sodium and placebo or within menstrual cycle phases (Table 3). AVP was significantly lower with sodium compared to placebo (−10.8 [−19.2, −2.3] pg·mL^−1^; *p* = 0.013), but this was observed only in the early-follicular phase (Figure 3). Plasma osmolality (8 [4.0, 12.0] mOsm·kgH_2_O^−1^; *p* < 0.001) and blood sodium (4 [3.0, 5.0] mmol·L^−1^; *p* < 0.001; Figure 4a,c) were significantly higher with sodium compared to placebo, and this was observed in both menstrual cycle phases. Plasma volume decreased less during TT (T+200 kJ) with sodium compared to placebo (6.38 [4.49, 8.28]%, *p* < 0.001; Figure 4b), and this was observed in both menstrual cycle phases. TT completion time was faster following sodium compared to placebo (22.41 [19.52, 25.31] vs. 23.95 [21.06, 26.85] min; *p* = 0.001). When analysed by menstrual cycle phase, TT time was shorter with sodium during the mid-luteal phase (22.06 [19.10, 25.02] vs. 23.91 [20.93, 26.89] min; *p* = 0.005) and showed a similar trend in the early-follicular phase (22.75 [19.79, 25.72] vs. 23.99 [21.03, 26.96] min; *p* = 0.054).

### 3.6. Sensitivity Analysis

A sensitivity analysis was performed excluding four participants (potential LPD group) who exhibited low progesterone concentrations (<16 nmol·L^−1^ during the mid-luteal phase; Supplementary Tables S1 and S2). The only differences compared to the main results include a lower percentage of body mass loss during the TT in the mid-luteal phase (*p* = 0.033) and intervention × menstrual cycle phase significant interactions for fluid retention (mL) and percentage of ingested fluid during the pre-exercise hyperhydration period (both *p* < 0.04). 

## 4. Discussion

This study provides new insights into the effects of pre-exercise sodium hyperhydration on hydration responses in female athletes, both at rest and during exercise in the heat, while also comparing physiological outcomes between the early-follicular and the mid-luteal phase of the menstrual cycle. Key findings indicate that pre-exercise sodium hyperhydration significantly improved hydration at rest, and this was observed in both menstrual cycle phases compared to placebo. Overall, sodium hyperhydration increased body mass at rest and during cycling, and reduced body mass loss and sweat rates during steady-state cycling compared to placebo. Exploratory analyses showed that reductions in body mass loss and sweat rates during the steady-state were significant only in the early-follicular phase, suggesting potential menstrual cycle phase-dependent differences in thermoregulatory responses to sodium hyperhydration and exercise. These physiological responses likely contributed to the lower AVP concentration observed post-TT with sodium hyperhydration in the early-follicular phase.

Our results extend previous sodium hyperhydration research in females; prior work examined exercise capacity (TTE) at a fixed intensity (70% VO_2peak_) [16], but not responses to higher-intensity self-paced exercise and did not include menstrual cycle phase comparisons. The same group [45] also assessed menstrual cycle phase differences (high vs. low hormone phases in oral contraceptive pill users and natural menstruating females) following the ingestion of an acute sodium chloride/citrate beverage (164 mmol·L^−1^ Na^+^, 10 mL·kg^−1^ body mass, ingested in seven portions over 60 min), but only under resting conditions and without evaluating hydration responses to exercise. The current investigation demonstrates for the first time that sodium hyperhydration effectively increases fluid retention at rest and improves hydration status during exercise across the menstrual cycle, with an attenuated body water loss during exercise in the early-follicular phase likely due to lower sweat losses. These findings suggest that it may be valuable to monitor hydration responses across menstrual cycle phases when developing hydration strategies for female athletes.

### 4.1. Responses at Rest

Overall, these findings confirm that pre-exercise sodium hyperhydration is an effective strategy to enhance fluid retention and mitigate dehydration of female athletes during endurance exercise in the heat. However, hydration responses may vary with menstrual cycle phase and exercise intensity. In our study, the ingestion of 7.5 g·L^−1^ of sodium chloride in gelatine pills with 30 mL·kg^−1^ fat-free mass of water two hours before exercise significantly enhanced fluid retention, as evidenced by increased plasma osmolality (~4 mOsm·kgH_2_O^−1^), higher USG (~0.002 au), reduced urine volume (~37%, equivalent to107.4 mL), and increased plasma volume (3.62%) compared to placebo. These findings align with previous research. In male participants, the ingestion of half the sodium dose used in our study (~3 g·L^−1^ of sodium, from sodium chloride) resulted in ~50% (~650 mL) lower urine volume compared to glycerol and water-induced hyperhydration after two hours at rest [11]. Likewise, in endurance-trained female cyclists, ingestion of 3.7 g·L^−1^ of sodium (from sodium citrate and sodium chloride) reduced urine volumes by 30% (~330 mL) compared to equivalent fluid intake with 0.2 g·L^−1^ of sodium (from sodium chloride) during the high-hormone phase of both naturally menstruating and oral contraceptive pill users [16]. In this study, sodium hyperhydration at rest (T-120 to T0) led to ~507 mL greater fluid retention and ~37% increase in the percentage of fluid retained compared to placebo. However, this increased fluid retention was not immediately reflected by changes in body mass over time (measured every 30 min), except during the mid-luteal phase. Several hydration markers, including fluid retention (603 vs. 415 mL), ingested fluid retained (41 vs. 30%), urine osmolality (45 vs. 22 mOsm·kgH_2_O^−1^), urine volume (−130 vs. −85 mL), urine sodium (600 vs. 303 ppm), plasma volume change (5.1 vs. 2.2%), and blood sodium (3 vs. 1 mmol·L^−1^), were higher with sodium compared to placebo in the mid-luteal phase compared to the early-follicular phase; however, these differences were not statistically significant. Although the absolute change in hydration markers, such as USG (~0.002 au), appears small, it indicates a measurable improvement in fluid conservation at rest. Participants were already euhydrated at baseline; therefore, the relevance of this enhancement lies not in correcting dehydration, but in increasing the pre-exercise body water content. Even modest pre-exercise fluid retention (~500 mL, equivalent to approximately 3–4% of plasma volume in a ~60–65 kg female athlete) can expand plasma volume before exercise begins, thereby improving cardiovascular stability, preserving stroke volume, and supporting skin blood flow and sweating responses during prolonged exercise in the heat.

Sensitivity analysis, which excluded participants with low progesterone concentrations, further amplified these differences. Fluid retention with sodium hyperhydration increased an additional ~73 mL (+5%), with a more pronounced effect in the mid-luteal phase (~141 mL; +14%), indicating that sodium hyperhydration has a more pronounced impact on hydration markers at rest in the mid-luteal phase. This may be attributed to higher progesterone levels, which can promote fluid retention via increased aldosterone sensitivity [46]. Although this increase in fluid retention may influence the power-to-weight ratio in weight-sensitive sports, such as uphill running and mountain cycling, it could also provide thermoregulatory advantages during exercise, including increased plasma volume and improved heat dissipation, which may offer a small but physiologically relevant advantage during prolonged efforts or in hot environments. During exercise, the mid-luteal phase is associated with higher thermoregulatory thresholds for sweating and cutaneous vasodilation [47,48,49], as well as an elevated osmoregulatory set-point, requiring higher plasma osmolality to trigger AVP release and thirst mechanisms [50]. These hormonal fluctuations across the menstrual cycle can alter electrolyte balance and hydration status [19,20], potentially modifying the responses to sodium hyperhydration.

Overall, the findings support established mechanisms of hydration regulation in females across the menstrual cycle, demonstrating that pre-exercise sodium hyperhydration enhances fluid retention at rest, with trends indicating variation across menstrual cycle phases. These results emphasise the importance of considering hormonal fluctuations across the menstrual cycle, which may impact fluid balance and thermoregulation in female athletes.

### 4.2. Responses During Exercise

At the end of steady-state cycling (T+75), body mass loss was significantly lower with sodium hyperhydration compared to placebo (−1.38% vs. −1.58%). This suggests that the fluid retained at rest (~509 mL; ~37% of ingested fluid) with sodium hyperhydration effectively delayed dehydration, reducing body mass loss by 0.2% after 75 min of steady-state exercise in the heat. This reduction in body mass loss is supported by higher plasma osmolality and blood sodium levels, as well as a smaller decrease in plasma volume during steady-state exercise with sodium hyperhydration compared to placebo. Although participants in both conditions maintained body mass loss below the 2% threshold typically associated with performance and thermoregulatory impairments, the smaller reduction with sodium hyperhydration indicates improved fluid conservation, which may help sustain plasma volume and cardiovascular stability during prolonged exercise in the heat [3], particularly when fluid access is limited. Similar responses have been observed at rest in male athletes, where sodium hyperhydration increased pre-exercise fluid retention by ~45%, leading to a 4.5% increase in plasma volume [51]. However, during treadmill running TTE at 70% VO_2max_, males lost more body mass overall despite the longer exercise duration. This suggests that the effectiveness of sodium hyperhydration may be influenced by exercise modality, intensity, and sex-specific physiological factors.

The menstrual cycle phase may further modulate hydration responses. Notably, the differences between sodium and placebo in body mass loss, sweat rate and sweat sodium concentration were smaller in the mid-luteal phase, whereas plasma volume changes were greater compared to the early-follicular phase. During the early-follicular phase, the osmotic threshold for AVP release and thirst is higher, promoting greater free water clearance and lower resting plasma volume compared to the mid-luteal phase [52,53]. Because of this lower baseline fluid retention, sodium loading may elicit a stronger compensatory response, with greater renal sodium and water conservation to restore osmotic balance. In contrast, elevated oestrogen and progesterone concentrations during the mid-luteal phase lower the osmotic threshold for AVP, stimulate sodium and water retention, and reduce the relative benefit of additional sodium ingestion [21,52,53]. Together, these mechanisms suggest that female athletes in the early-follicular phase, when sex hormone concentrations and baseline fluid retention are lower, may be more responsive to the hydration benefits of sodium hyperhydration, allowing greater plasma volume expansion and delayed dehydration during moderate-intensity exercise in the heat.

The improved hydration status observed with sodium hyperhydration persisted throughout the TT. By the end of TT, female athletes maintained ~0.8 kg higher body mass and experienced lower total body mass loss (~0.8%) with sodium compared to placebo. Plasma volume change, plasma osmolality, and blood sodium concentration also remained elevated with sodium hyperhydration. However, unlike during steady-state exercise, overall sweat rates during the TT did not differ between sodium and placebo, suggesting that during self-paced, high-intensity exercise, the body’s thermoregulatory drive to sweat may override the effects of pre-exercise sodium hyperhydration [54]. Nevertheless, the greater plasma volume and osmotic stability likely supported cardiovascular and thermoregulatory function under heat stress [55], contributing to the faster TT completion times observed with sodium hyperhydration. By the end of the TT, AVP levels were significantly lower with sodium compared to placebo, likely due to the improved hydration status with sodium hyperhydration attenuating the hormonal drive to conserve water [21,56].

Since total body mass losses after exercise with sodium hyperhydration were similar, the reduced AVP response in the early-follicular phase suggests that females may be less sensitive to hormonal-mediated body water conservation when ovarian hormone levels are lower. Conversely, in the mid-luteal phase, when ovarian hormones are elevated, females may be more susceptible to body water conservation due to elevated AVP levels despite a reduced body mass loss with sodium hyperhydration and exercise. Notably, although water conservation during steady-state work appeared more evident in the early-follicular phase, the larger TT benefit occurred in the mid-luteal phase of the menstrual cycle. One possible interpretation is that sodium hyperhydration may offset the greater thermal and cardiovascular strain typically present in the mid-luteal phase [49] (e.g., higher thermoregulatory thresholds and altered osmoregulatory set-points), allowing athletes to sustain higher power output when the physiological load is otherwise greatest.

At rest, fluid retention with sodium hyperhydration is more pronounced in the mid-luteal phase. In contrast, during steady-state exercise, reduced sweat rate and body mass loss may support improved thermoregulation and hydration status in the early-follicular phase. Following prolonged, high-intensity exercise, AVP responses may remain elevated despite sodium hyperhydration, while improved hydration status appears more pronounced in the mid-luteal phase. The hydration and osmoregulatory advantages observed with sodium hyperhydration are consistent with, and likely contribute to, the improved self-paced performance in this cohort. Importantly, these findings also suggest that the mid-luteal phase may pose a greater risk for exercise-associated hyponatremia in susceptible individuals [57], as elevated progesterone levels in this phase promote water retention and alter osmoregulatory thresholds, potentially leading to a dilution of plasma sodium. To prevent this, athletes should consume sodium-containing beverages during exercise to maintain euhydration [57,58] and preserve plasma sodium concentrations, mitigating the risk of exercise-associated hyponatremia. Together, these results underscore the importance of considering menstrual cycle phase when developing individualised hydration strategies for female athletes.

## 5. Limitations

Participants did not consume any fluid during exercise, limiting the ability to assess the impact of voluntary, ad libitum fluid intake. Previously published data [24] indicated no differences in thirst sensation with sodium hyperhydration pre-exercise and during TT, but a reduction in thirst was observed during steady-state compared to placebo. While this reduced thirst sensation with sodium hyperhydration may help mitigate the risk of overhydration and, in consequence, hyponatraemia during steady-state exercise, it could also compromise the maintenance of optimal hydration status during prolonged exercise. Additionally, although some evidence suggests that ad libitum sodium intake may vary across menstrual cycle phases [21], in the present study, dietary intake was replicated and controlled to standardise baseline hydration status. While this approach strengthens internal validity, it may limit the generalisability of these findings to real-world scenarios where athletes self-regulate fluid and sodium intake. Furthermore, these results are specific to the controlled laboratory conditions of this study and may not fully translate to varied exercise settings or hormonal profiles of hormonal contraceptive users and/or women across the menopausal transition.

It should also be acknowledged that plasma AVP was quantified directly rather than using copeptin, the more analytically stable *C*-terminal fragment of the pre-pro-vasopressin precursor [59,60]. AVP quantification was selected to capture the biologically active hormone mediating renal water reabsorption and vascular tone via V1a, V1b, and V2 receptors [60,61], and to align with previous menstrual-cycle studies examining fluid dynamics [21]. While copeptin strongly correlates with AVP secretion and offers greater assay stability, it reflects precursor release rather than the circulating active peptide. Future studies may include both markers to provide complementary insights into precursor secretion and active hormone dynamics [59,62].

While the study was not specifically powered to detect differences between participants with and without LPD, these comparisons were exploratory and secondary to the primary aims. As such, the sample size may limit definitive conclusions of subgroup differences, particularly with distinct hormonal profiles. Future research with larger cohorts is needed to explore more complex interactions across the menstrual cycle, differentiate between the effects of endogenous and exogenous hormones and better understand their impact on hydration and exercise performance outcomes.

## 6. Conclusions

Pre-exercise sodium hyperhydration is an effective strategy for enhancing fluid retention and mitigating dehydration in female endurance athletes exercising in the heat. The benefits of sodium hyperhydration persist across fixed-intensity and self-paced high-intensity efforts, with some indications that menstrual cycle phase may influence hydration markers and AVP. Female athletes can implement sodium hyperhydration (~7.5 g·L^−1^ sodium with 30 mL·kg^−1^ fluid intake) approximately two hours before exercise to optimise hydration responses during endurance exercise in the heat, particularly when fluid access is limited. Rather than prescribing phase-specific strategies, it may be valuable for athletes and practitioners to monitor hydration responses across menstrual cycle phases, given the potential for individual variability in fluid retention and sweat losses. Ultimately, individualised hydration strategies that consider exercise intensity, environmental conditions, and individual responses are recommended for optimising fluid balance, thermoregulation, and endurance performance in female athletes.

## Figures and Tables

**Table 1:** Comparison of participants’ baseline characteristics between no luteal phase deficient and potential luteal phase deficient participants.

	No-LPD (*n* = 8)	LPD (*n* = 4)
	Mean ± SD	Mean ± SD
Age (years)	35 ± 5	31 ± 1
Height (cm)	167 ± 7	160 ± 1
Body mass (kg)	61.7 ± 6.6	63.5 ± 6.1
Fat-free mass (kg)	46.2 ± 4.9	46.6 ± 4.0
Body fat (%)	27.1 ± 4.8	27.3 ± 1.2
BSA (m^2^)	6.9 ± 0.5	6.8 ± 0.5
VO_2peak_ (mL·kg^−1^ BM·min^−1^)	56.6 ± 5.6	47.2 ± 8.7

No-LPD: No luteal phase deficiency group; LPD: potential luteal phase deficiency subgroup (excluded from the sensitivity analysis); BSA: body surface area.

**Table 2:** Physiological outcomes at baseline and in response to a two-hour hyperhydration intervention at rest with sodium and placebo across the early-follicular and mid-luteal phases of the menstrual cycle.

		Baseline				Pre-Exercise (2 h at Rest)			
		Sodium	Placebo	Difference *p*-Value	Interaction *p*-Value	Sodium	Placebo	Difference *p*-Value	Interaction *p*-Value
		Hydration Outcomes							
Fluid retention (mL)	Overall	-	-	-	-	616.1 (443.5, 788.8)	109.2 (−65.4, 283.7)	<0.001	-
	EF	-	-	-	-	568.3 (362.7, 774)	153.1 (−52.6, 358.8)	<0.001	0.252
	ML	-	-	-		666 (460.3, 871.7)	63.3 (−149, 275.7)	<0.001	
Ingested fluid retained (%)	Overall	-	-	-	-	44.4 (31.8, 57.0)	7.6 (−5.1, 20.4)	<0.001	-
	EF	-	-	-	-	40.5 (25.5, 55.6)	10.8 (−4.2, 25.9)	<0.001	0.230
	ML	-	-	-		48.5 (33.4, 63.5)	4.3 (−11.2, 19.9)	<0.001	
		Urine Outcomes							
Urine Specific Gravity (au)	Overall	1.015 (1.012, 1.018)	1.013 (1.010, 1.016)	0.340	-	1.007 (1.006, 1.008)	1.005 (1.004, 1.006)	<0.001	-
	EF	1.014 (1.010, 1.018)	1.013 (1.009, 1.017)	0.588	0.840	1.007 (1.006, 1.008)	1.005 (1.004, 1.006)	0.021	0.729
	ML	1.016 (1.012, 1.019)	1.014 (1.010, 1.018)	0.420		1.008 (1.006, 1.009)	1.005 (1.004, 1.007)	0.005	
Urine osmolality (mOsm·kgH_2_O^−1^)	Overall	556 (436, 676)	483 (359, 608)	0.207	-	298.5 (255.6, 341.3)	206.2 (163.1, 249.3)	<0.001	-
	EF	541 (400, 683)	481 (335, 628)	0.448	0.829	288.5 (236.5, 340.5)	207.7 (156.3, 259.1)	0.007	0.604
	ML	570 (428, 712)	486 (334, 637)	0.301		308.7 (257.6, 359.8)	204.6 (151.9, 257.3)	0.001	
Urine Volume (mL)	Overall	49.7 (32.4, 67.1)	51.6 (32.7, 70.5)	0.874	-	185.4 (144.6, 226.2)	293.1 (251.9, 334.3)	<0.001	-
	EF	54.1 (30.9, 77.3)	60.4 (35, 85.8)	0.705	0.722	204.4 (154.9, 253.8)	289.9 (240.7, 339.2)	0.003	0.257
	ML	45.3 (22.1, 68.5)	43.3 (17.9, 68.6)	0.902		166.9 (118.2, 215.7)	296.3 (246, 346.7)	<0.001	
Urine Sodium (ppm)	Overall	1525 (1139, 1910)	1274 (868, 1680)	0.255	-	989.4 (821.6, 1157.1)	530.3 (360.8, 699.9)	<0.001	-
	EF	1344 (871, 1817)	1408 (917, 1899)	0.830	0.127	891.5 (680, 1103)	586.3 (376.6, 796)	0.020	0.109
	ML	1705 (1232, 2178)	1123 (611, 1635)	0.057		1090 (881.5, 1298.5)	473.8 (257.6, 690)	<0.001	

*n* = 12. Results are presented as mean and 95% confidence intervals (CI) for overall and phase effects estimated under a linear mixed model, including ‘intervention’, ‘phase’, and ‘time’ (categorical) and all possible interactions as fixed effects. Baseline corresponds to T-120; pre-exercise corresponds to T-120 until T0 (rest). Overall: comparison between sodium and placebo regardless of phase; EF, early follicular phase: low oestrogen and progesterone; ML, mid-luteal phase: moderate oestrogen and high progesterone.

**Table 3:** Physiological outcomes in response to two-hour hyperhydration intervention with sodium and placebo after 75 min of steady-state and 200 kJ time trial (TT) cycling across early-follicular and mid-luteal phases of menstrual cycle.

		Steady State Cycling				Cycling Time Trial			
		Sodium	Placebo	Difference *p*-Value	Interaction *p*-Value	Sodium	Placebo	Difference *p*-Value	Interaction *p*-Value
		Hydration Outcomes							
Body Mass Loss (%)	Overall	1.38 (1.21, 1.54)	1.58 (1.41, 1.75)	0.001	-	0.77 (0.59, 0.96)	0.91 (0.73, 1.1)	0.037	-
	EF	1.31 (1.12, 1.5)	1.57 (1.38, 1.76)	0.004	0.395	0.82 (0.61, 1.03)	0.92 (0.71, 1.13)	0.332	0.466
	ML	1.44 (1.25, 1.63)	1.59 (1.4, 1.79)	0.105		0.72 (0.51, 0.92)	0.91 (0.7, 1.13)	0.050	
Sweat Rate (L·h^−1^)	Overall	0.60 (0.52, 0.68)	0.68 (0.61, 0.76)	0.001	-	2.09 (1.80, 2.37)	2.12 (1.84, 2.41)	0.739	-
	EF	0.57 (0.49, 0.66)	0.67 (0.58, 0.75)	0.003	0.457	2.16 (1.84, 2.48)	2.12 (1.79, 2.44)	0.788	0.479
	ML	0.64 (0.55, 0.72)	0.70 (0.61, 0.79)	0.062		2.01 (1.68, 2.33)	2.13 (1.80, 2.46)	0.468	
Sweat Sodium (ppm)	Overall	1530 (1316, 1744)	1430 (1215, 1646)	0.109	-	-	-	-	-
	EF	1551 (1321, 1782)	1433 (1203, 1663)	0.169	0.754	-	-	-	-
	ML	1507 (1277, 1737)	1427 (1194, 1661)	0.368		-	-	-	

*n* = 12. Results are presented as mean and 95% confidence intervals (CI) for overall and phase effects estimated under a linear mixed model including ‘intervention’, ‘phase’, and ‘time’ (categorical) and all possible interactions as fixed effects. Steady state corresponds to T+75; Time trial corresponds to T+200 kJ. Overall: comparison between sodium and placebo regardless of phase; EF, early follicular phase: low oestrogen and progesterone; ML, mid-luteal phase: moderate oestrogen and high progesterone.

## Data Availability

The original contributions presented in this study are included in the article. Further inquiries can be directed to the corresponding author.

## Supplementary Materials

Supplementary Tables S1 and S2 are available at: https://www.doi.org/10.6084/m9.figshare.28765721.
